# Tomato Genome-Wide Transcriptional Responses to Fusarium Wilt and Tomato Mosaic Virus

**DOI:** 10.1371/journal.pone.0094963

**Published:** 2014-05-07

**Authors:** Giuseppe Andolfo, Francesca Ferriello, Luca Tardella, Alberto Ferrarini, Loredana Sigillo, Luigi Frusciante, Maria Raffaella Ercolano

**Affiliations:** 1 Department of Agriculture Sciences, University of Naples ‘Federico II’, Portici, Italy; 2 Department of Statistical Sciences, University of Rome ‘La Sapienza’, Rome, Italy; 3 Dipartimento di Biotecnologie - Università degli Studi di Verona, Strada le Grazie, Verona, Italy; 4 Consiglio per la Ricerca e Sperimentazione in Agricoltura - Centro di sperimentazione e certificazione delle sementi (CRA-SCS) S.S., Battipaglia (SA), Roma, Italy; National Taiwan University, Taiwan

## Abstract

Since gene expression approaches constitute a starting point for investigating plant–pathogen systems, we performed a transcriptional analysis to identify a set of genes of interest in tomato plants infected with *F. oxysporum f. sp. lycopersici* (*Fol*) and Tomato Mosaic Virus (*ToMV*). Differentially expressed tomato genes upon inoculation with *Fol* and *ToMV* were identified at two days post-inoculation. A large overlap was found in differentially expressed genes throughout the two incompatible interactions. However, Gene Ontology enrichment analysis evidenced specific categories in both interactions. Response to *ToMV* seems more multifaceted, since more than 70 specific categories were enriched versus the 30 detected in *Fol* interaction. In particular, the virus stimulated the production of an invertase enzyme that is able to redirect the flux of carbohydrates, whereas *Fol* induced a homeostatic response to prevent the fungus from killing cells. Genomic mapping of transcripts suggested that specific genomic regions are involved in resistance response to pathogen. Coordinated machinery could play an important role in prompting the response, since 60% of pathogen receptor genes (**NB-ARC-LRR**, **RLP**, **RLK**) were differentially regulated during both interactions. Assessment of genomic gene expression patterns could help in building up models of mediated resistance responses.

## Introduction

Disease resistance in plants depends on the ability of the host to recognize pathogens and initiate defense mechanisms that limit infection. A basal type of immunity in plants is conferred by the recognition of conserved microorganism-associated molecular patterns (MAMPs) by specific pattern-recognition receptors (PRRs) that protect hosts against non-specialized pathogens. Plants are also capable of establishing immune responses by using pathogen receptors (mainly Nucleotide binding-ARC/leucine-rich repeat proteins **NB-ARC-LRR** but also Receptor Like Proteins **RLP**; Receptor-Like Kinase **RLK**) known as resistance (R) proteins able to recognize the presence of pathogen effector molecules and to activate effector-triggered immunity (ETI). This response is typically associated with programmed cell death of the infected cells and the production of antimicrobial molecules [1]. Modulation of hormone pathways is required to restrict pathogen invasion, re-allocate resources, control cell death and modify plant architecture [2]. In addition, systemic-acquired resistance, which immunizes against subsequent infections, could also occur [1].

Tomato (*Solanum lycopersicum*) has served as an important model system for studying the genetics and molecular basis of resistance mechanisms in plants. The breadth of pathogen classes affecting tomato underscores the importance of tomato pathosystems as amenable models for studying the plant immune system [3]. Plants build up appropriate defense responses without draining energy resources to unsustainable levels through the cross-talk and fine-tuning of different defense pathways [4]. Identification of host genes involved in resistance responses could be helpful both for understanding plant defence mechanisms against pathogens and building up a biological model of plant–pathogen interaction.

*Fusarium oxysporum* f. sp. *lycopersici* (*Fol*) is one of the main diseases affecting tomato, causing vascular wilt disease by colonizing the xylem vessels of roots and stems. Three physiological races, termed 1, 2 and 3, have been identified. The *FOL AVR* genes (*AVR1, AVR2* and *AVR3*) are carried in different combinations in different *FOL* races. *AVR1* ( = *SIX4*) is unique to race 1 whereas *AVR2* ( = *SIX3*) is found in races 1 and 2. *AVR3* ( = *SIX1*), which exists in all races *I*, *I2* and *I3* are known confer resistance to FOL in tomato. Race 1-resistant cultivars (*I i2 i3*), races 1 and 2-resistant cultivars (*I I2 i3*), and races 1, 2 and 3-resistant cultivars (*I I2 I3*) have been developed [5]. To date only I-2 gene, a coiled-coil/nucleotide binding-ARC/leucine-rich repeat (CC-NB-ARC-LRR) protein, that confers resistance to race 2 of *Fusarium oxysporum f. sp. lycopersici* has been cloned [6]; [7]. Responses mainly involves the callose deposition, the accumulation of phenolics and the formation of tyloses (outgrowths of xylem contact cells) and gels in the infected vessels [8]. Interaction between tomato and *Fusarium oxysporum f. sp.lycopersici* has been investigated in depth, becoming a model system for disease resistance response [9].

Tomato mosaic virus (ToMV) is a positive-sense ssRNA virus belonging to the Tobamovirus genus. ToMV infects tomato plants systemically, causing mosaic symptoms, which are characterized by intermingled light and dark green regions [10]. The Tm-2 gene of tomato and its allelic form, Tm-2(2), confer resistance to Tomato mosaic virus (ToMV) and encode members of the CC-NB-ARC-LRR protein class [11].

These two pathogens have different infection strategies: *ToMV* is a biotrophic leaf pathogen that causes tomato leaf mold, whereas *Fol* is a saprotrophic soilborne vascular pathogen that challenges several Solanaceae. The aim of this study was to compare tomato global transcriptional profiles in response to host attack by *ToMV* and *Fol* in order to identify genomic differences and similarities in incompatible interactions between a foliar and a vascular pathogen, making use of the recently sequenced tomato genome. First we examined global tomato transcriptional profiling during both incompatible interactions to compare transcriptional changes occurring in tomato plants. Then we performed in-depth gene annotation, highlighting enriched GO categories and any metabolic perturbations arising. Finally, we explored the genome arrangement of expressed genes along the chromosome in order to connect genomic and transcriptional events.

## Materials and Methods

### Plant Material and Inoculation Protocol

For our experiments we used the Rosso Delta tomato (*S.lycopersicum*) variety, resistant to *Fol* races 1 and 2 and *ToMV* race 0. Tomato plants were inoculated with *Fol* strain ATCC 16605 containing the Avr1 and Avr2, (Plant Research International, NL). Plantlets were infected at the stage of expanded cotyledons: roots were cut and dipped in a suspension at a concentration of 1×10^6^ conidia/ml, according to CPVO technical protocol TP/044/3 [12] (Experiment I). Inoculation with *Tomato Mosaic Virus* was carried out with the strain GM6s of *ToMV* (Plant Research International, NL), according to the method reported in the CPVO technical protocol TP/044/3 [12] (Experiment II). Plants with expanded cotyledons were inoculated with the sap obtained from infected desiccated tomato leaves. Viral transmission was ensured by mechanical tissue abrasion caused by diatomaceous powder.

### Sample Collection

Two days after treatment, infected and non-infected tomato leaf samples of two independent replicas of Experiment I and II were collected. Roots and leaves were removed from the plants, weighed and immediately frozen in liquid nitrogen and stored at −80°C. RNA was isolated from whole plants using the Rneasy Plant Kit according to the manual instructions (Quiagen Valencia, USA). RNA sample concentration was determined using a Nanodrop photometer. An Agilent 2100 Bioanalyzer was used to check the quality of RNA.

### Chip Design and Microarray Hybridization

Transcriptomic analysis was performed using a 90 K TomatArray 2.0 microarray synthesized using the CombiMatrix platform at the Plant Functional Genomics Center of the University of Verona. The chip (TomatoArray2.0) carries 25,789 non redundant probes (23,282 unique probes and 2,507 probes with more than one target) randomly distributed in triplicate across the array, each comprising a 35–40-mer oligonucleotide. The source of sequence information included tentative consensus sequences (TCs) derived from the DFCI Tomato Gene Index Release 12.0 and expressed sequence tags. Eight bacterial oligonucleotide sequences provided by CombiMatrix, eight probes designed on eight Ambion spikes and 40 probes based on *Bacillus anthracis*, *Haemophilus ducreyi* and *Alteromonas* phage sequences were used as negative controls. Complete description of the chip is available at the Gene Expression Omnibus under the platform accession GPL13934. Microarray analysis was used to investigate tomato gene expression profiles two days after the infection with *Fol* and *ToMV*, comparing with the uninfected control profile. After checking the quantity and quality by spectrophotometry using NanoDrop 1000 (Thermo Scientific), total RNA (2 µg) was amplified and labeled using the RNA ampULSe kit (Kreatech). For array hybridization, according to the manufacturer’s recommendations, 4 µg of labeled aRNA was employed. Cy5 labeled aRNA was hybridized with the microarray at 45°C for 16 h; then arrays were washed with hybridization Solution at 45°C for 5 minutes and 3×SS, 0.5×SSPET, PBST wash and PBS wash at room temperature. Pre-hybridization, hybridization, washing and imaging steps were carried out according to the manufacturer’s instructions. Microarrays were stripped for reuse (up to three reuses per chip) with the CombiMatrix CustomArrayTM Stripping Kit according to the manufacturer’s instructions (product number 610049). After hybridization and washing, the microarray was scanned using a Perkin Elmer Scan Array 4000XL (software ScanArray Express Microarray Analysis System v4.0).

### Data Analysis

Gene expression levels corresponding to eight microarrays were processed using R software (R Core Team 2013 [13], URL http://www.R-project.org/.) and the limma package [14]. Raw data were investigated for quality assessment, preprocessed and normalized using a suitable subset of control probes available in the CombiMatrix array and a quantile normalization technique. Three technical replicates within each array and two biological replicates were employed to assess differential expression for each experiment (Experiments 1 and 2) to compare the different experimental conditions (non-inoculated vs inoculated) using a linear model for microarray [15]. The significance of the differential expression was assessed taking into account the multiple testing setting and controlling the False Discovery Rate (FDR) at FDR = 0.01 and the empirical Bayes moderated t-statistics available in the limma package. Linear model fitting was used to assess differential expression of the two pairs of contrasted experimental conditions (non-inoculated vs inoculated), taking into account the presence of technical replicates as well as biological replicates. Differential expression data are deposited in Gene Expression Omnibus (**GEO**) database repository with accession number (GSE52336).

### Annotation of the Gene Chip Probes

An in-house pipeline was developed to annotate tomato tentative consensus sequences (TCs) used to develop CombiMatrix CustomArrayTM probes. The queried tomato genes were identified by mapping TC sequences to the tomato CDS sequence using BLASTn (E-value 1e-3). The latest version of the tomato gff3 annotation files was parsed to extract the cds sequences of genes probed. Blast2GO http://blast2go.bioinfo.cipf.es/was used to provide automatic high-throughput annotation, gene ontology mapping and categorization of tomato proteins identified. An expectation value threshold of 1e-6 in BLASTp analysis was performed. Blast2GO was used for the statistical analysis of GO-term frequency differences. The enrichment analysis of the GO terms was based on Fisher’s exact test and corrects for multiple testing. A cut-off FDR value of 0.05 was used. An interactive graph of the GO category enrichment REVIGO htt://revigo.org/and a graphic representation Cytoscape htt://cytoscape.org//were also performed.

## Results

### Identification of Differentially Expressed Genes Induced by *F. oxysporum* f. sp. *lycopersici* (*Fol*) and *ToMV* Inoculation

The transcriptional responses of resistant tomato seedlings, inoculated with *Fol* and ToMV, were evaluated by querying 15,734 tomato genes. This analysis allowed us to compare changes occurring during both incompatible interactions with a vascular fungus and with a virus. Differentially expressed tomato genes (FDR-adjusted p-value <0.01) were identified at 2 DPI, comparing inoculated and non-inoculated plants. The time point was selected in order to identify genes involved in the initial stages of the defense against the pathogens.

**Figure 1:**
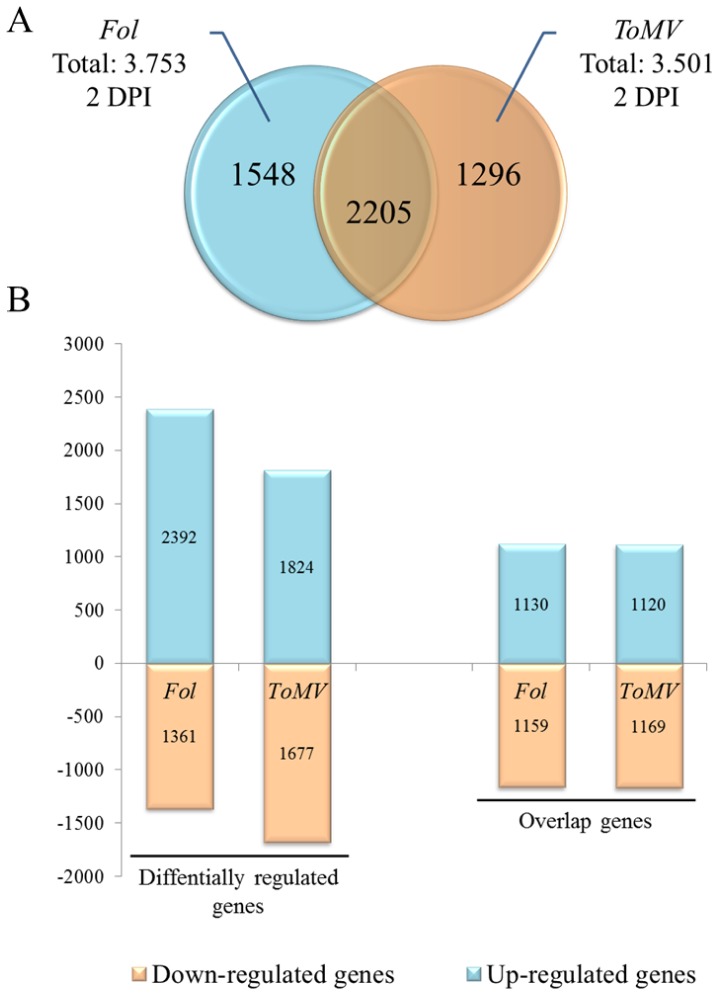
Comparison of tomato differentially regulated genes during interaction with *Fusarium oxysporum f. sp. lycopersici (Fol)* and Tomato Mosaic Virus *(ToMV)* at 2 DPI post-inoculation. **A**, Venn diagram of differentially expressed genes in *Fol* and *ToMV* tomato incompatible interactions. **B**, Histogram of up regulated and down regulate genes in tomato-*Fol* interaction and tomato-*ToMV* interaction.

In *Fol* incompatible interaction, transcriptome variation resulted in 3,753 differentially induced genes. In particular, 2,392 genes (about 64%) were up-regulated (Fig. 1A), indicating considerable gene activation during infection. As for the vascular pathogens, 3,501 transcriptional changes were monitored in tomato upon inoculation with *ToMV,* of which about 2000 (52%) were overexpressed. When the total number of differentially regulated genes between the two incompatible interactions were compared, roughly half (2,205 genes) overlapped, of which a small subset of 131 genes had the opposite expression direction during the two interactions. As shown in Fig. 1B, a marked host gene induction was evident, since more than half of the differentially expressed genes appeared to be induced in both interactions. Transcriptional changes during the two tomato-pathogen interactions were further investigated in order to find the best strategy for building a biological model for a plant-pathogen system.

### Gene Set Enrichment Analysis

We performed a GO-term annotation analysis of all transcripts identified by gene chip probe matching. Through this analysis, we were able to assign functional annotations to 15,378 chip transcripts. In order to facilitate the formulation of biological hypotheses, a categorization of gene expression data was performed. Indeed, the Gene Ontology research effort was extremely useful for structuring data description.

**Figure 2:**
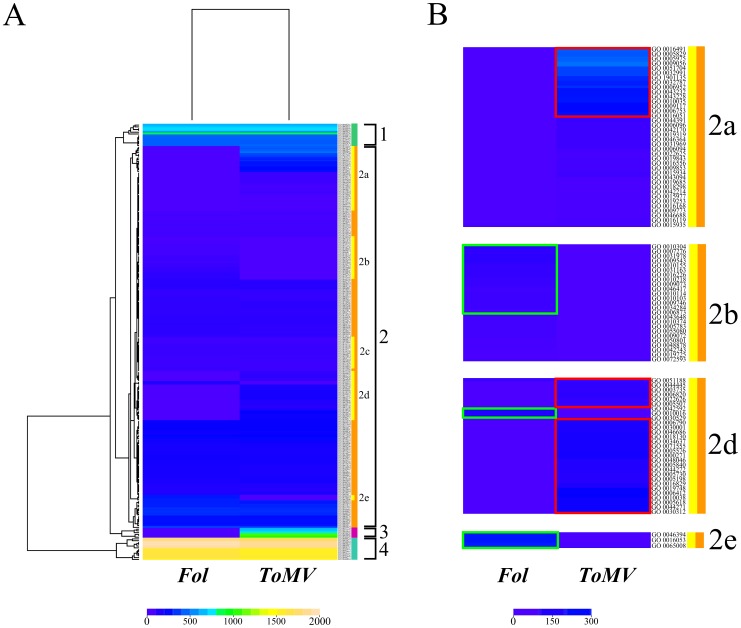
Heatmap of Gene Ontology enriched terms in the two interactions. Gene Ontology terms enriched are obtained comparing a differential group (differentially regulated genes in each interaction) whit a reference group (all gene of chip) using Fisher’s Exact Test, with Multiple Testing Correction of FDR, estimated at P value of <0.05. **A**, The rows of heatmap represent GO-terms and the columns represent samples. Each cell is colorized based on the number of genes associated with that category GO in that particular sample. **B**, Subclusters composed of specific GO-terms of the tomato-pathogen interactions. The green boxes highlight specific GO terms of tomato-*Fol* interaction, the red boxes specific GO terms of tomato-*ToMV* interaction.

In the tomato-*ToMV* interaction 226 enriched GO terms were detected, 70% of which belonged to a biological process (P) category, while in the fungus interaction fewer enriched categories (185) were identified, 78% of which belonged to a biological process (P) category (Fig. 2A). Comparing the total number of GO categories between the two incompatible interactions, roughly 60% overlapped.

A heatmap obtained through hierarchical analysis evidenced four specific GO term clusters. Clusters 1 and 4 included GO ontology terms that did not show significant differences in two experiments. Cluster 2 contained about 90% of the enriched GO terms. Subcluster 2b consisted of specific GO terms of tomato-*Fol* interaction, whilst subclusters 2a and 2d as well as cluster 3 included tomato-*ToMV* interaction specific ontology terms (Fig. 2B).

**Figure 3:**
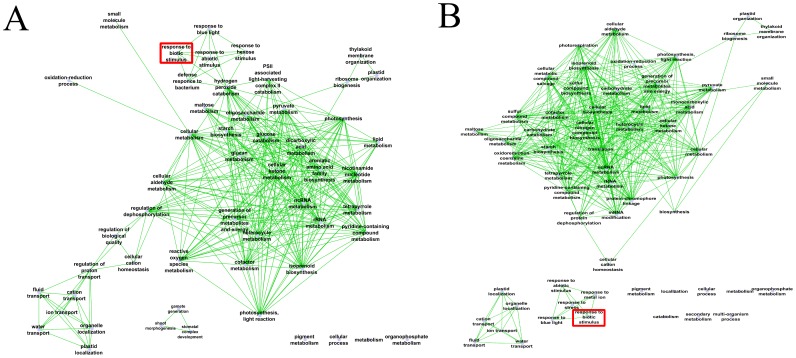
The interactive graph-based enriched gene-ontology (A) in fungus-tomato interaction and (B) in virus-tomato interaction.

To reduce redundancy of functional categories between the enriched GO terms, we used the semantic similarity approach. The gene ontology interactive graph-based network produced (Fig. 3) encapsulated functional homology between genes of the two tomato-pathogen interactions. In the tomato-*Fol* interaction, the network comprised 53 nodes and 310 edges, while for the tomato-*ToMV* interaction the network was more complex (58 nodes and 383 edges).

The network of tomato-*Fol* interaction showed that the biotic stimulus node (GO:0009607) correlated with the five internal nodes with gene ontology terms related to external stimuli response (GO:0009637, GO:0009746, GO:0009628, GO:0042742, GO:0042744). The GO:0009607 category includes a large number of genes that play a role in signal transduction and regulation of gene expression in the defense response, representing about 10% of differentially expressed genes in tomato-*Fol* interaction. The external stimuli sub-network was also connected with hydrogen peroxide catabolism node (GO:0042744). It contained 28 genes, 70% of which were overexpressed. In particular, four haem peroxidases (Solyc01g006290.2.1; Solyc01g006300.2.1; Solyc06g050440.2.1; Solyc11g018800.1.1), which play a role in host defense by inhibiting the hyphal extension of invading pathogens, were included in this node.

Fig. 3B shows the network obtained with significant GO-term changes in tomato-*ToMV* interaction. The biotic stimuli GO:0009607 node correlated with four GO-term nodes (GO:0009637, GO:0006950, GO:0010038, GO:0009628), associated to 264 genes. Interestingly, no external link was evidenced for this interaction. These data indicate that incompatible interactions induced both well documented stress-responsive genes as well as unknown genes that might play a role in multi-stress responses. Investigation of single GO categories could evidence GO terms related to each assessed specific response.

### Investigation of Perturbed Biological Processes

To obtain an overview of processes involved in fungus and virus incompatible reactions in-depth GO (gene ontology) term analysis was performed. Significant differences were observed in specific regulated biological processes between the two incompatible interactions. Notably, 30 specific enriched GO-term categories for tomato-*Fol* interaction, and 71 for tomato-*ToMV* interaction were identified (Table S1and S2 in File S1).

Tomato-*Fol* interaction revealed changes in cell structure, light perception, iron transport, aromatic amino acid synthesis and ROS response. In particular, we found six specific GO terms associated to homeostatic process (GO:0006873 cellular ion homeostasis; GO:0055080 cation homeostasis; GO:0050801 ion homeostasis; GO:0019725 cellular homeostasis; GO:0048878 chemical homeostasis and GO:0042592 homeostatic process) that included 104 genes. This strongly suggests that homeostasis plays an important role in tomato defense to *Fol*. Interestingly, the master gene of inflammation, NF-kB (Solyc02g094530.1.1) was also up-regulated in tomato-*Fol* interaction. This gene is a key player in anti-apoptotic signaling and is able to prevent apoptotic signaling by inhibiting map-kinases. Indeed, tomato map–kinases (Solyc12g019460.1.1; Solyc11g072630.1.1) were found to be heavily down-regulated during this interaction. Table 1 shows 14 auxin-binding genes and two abscisic acid receptors up-regulated in tomato-*Fol* interaction.

**Table 1:** List of abscisic acid receptors, auxin response factors (ARFs) and auxin response genes up-regulated in the tomato-*Fol* interaction.

Gene ID	Probe ID	Putative function	GO-ID terms
**Solyc01g091030.2.1**	TC196630	Auxin responsive SAUR protein	GO:0009733; GO:0009862; GO:0003674
**Solyc02g077560.2.1**	TC192465	Auxin response factor	GO:0009725; GO:0009734; GO:0009850; GO:0009733
**Solyc03g007310.2.1**	TC197392	Abscisic acid receptor	GO:0010427
**Solyc03g031970.2.1**	TC207784	Auxin response factor	GO:0009734
**Solyc03g120500.2.1**	TC199757	Auxin responsive protein	GO:0009734
**Solyc04g074980.2.1**	TC207543	Auxin F-box protein	GO:0009734
**Solyc05g047460.2.1**	TC205766	Auxin response factor	GO:0009734
**Solyc06g061180.1.1**	TC193498	Abscisic acid receptor	GO:0010427; GO:0009738
**Solyc07g016180.2.1**	TC207832	Auxin response factor	GO:0009734
**Solyc07g043610.2.1**	TC207520	Auxin response factor	GO:0003677; GO:0009734
**Solyc07g063850.2.1**	TC216744	GH3 auxin-responsive promoter	GO:0010252; GO:0009734
**Solyc09g014380.2.1**	TC216697	Auxin transporter-like protein	GO:0009734; GO:0010328; GO:0060919; GO:0009926; GO:0010011
**Solyc10g050710.1.1**	TC202297	GH3 auxin-responsive promoter	GO:0010279; GO:0009733
**Solyc10g083320.1.1**	TC194758	Auxin responsive SAUR protein	GO:0009733
**Solyc11g069190.1.1**	TC208143	Auxin response factor	GO:0009734
**Solyc12g005310.1.1**	TC203007	GH3 auxin-responsive promoter	GO:0010279; GO:0010279; GO:0009734

For each entry is reported: Gene and probe ID, description of putative function and GO- ID term correlated.

Photosynthetic and carbohydrate derivative processes, nitrogen biosynthesis, therpene and carotenoid metabolism, and cadmium/copper function were challenged in tomato-*ToMV* interaction. Three specific GO terms associated to photosynthetic processes (GO:0009853 photorespiration; GO:0019685 photosynthesis dark reaction and GO:0009773 photosynthetic electron transport in photosystem I) were identified and were investigated further. Table 2 shows the enriched categories associated to photosynthetic processes containing 191, genes over 86% of which were down-regulated. Interestingly, photosynthetic gene transcription repression was inversely correlated with the pathogenesis-related gene induction (Table 3). Moreover, a close linkage clearly emerged between the plant carbohydrate status and the resulting plant pathogen interaction. We found 17 carbon fixation enzymes differentially expressed during tomato-*ToMV* interaction. Furthermore, several genes involved directly in the Calvin cycle were down-expressed (Table 4), and β-fructofuranosidase (Solyc10g085640.1.1), a key invertase involved in sink source portioning, was overexpressed. We also found up-regulated six gibberellin-modulated genes and two genes involved in SA (Solyc01g014320.2.1) and JA (Solyc07g042170.2.1) synthesis (Table 5).

**Table 2:** List of the GO-terms involved in the photosynthetic process during the tomato-*ToMV* interaction.

GO ID	Term	Category	n. genes	% downeregulated genes
**GO:0009521**	photosystem	cellular component	25	100
**GO:0009522**	photosystem I	cellular component	16	100
**GO:0009523**	photosystem II	cellular component	18	100
**GO:0009765**	photosynthesis, light harvesting	biological process	17	100
**GO:0009767**	photosynthetic electron transport chain	biological process	24	100
**GO:0009773**	photosynthetic electron transport in photosystem I	biological process	15	86,7
**GO:0010207**	photosystem II assembly	biological process	50	94
**GO:0015979**	photosynthesis	biological process	125	91,2
**GO:0019684**	photosynthesis, light reaction	biological process	93	94,6
**GO:0019685**	photosynthesis, dark reaction	biological process	12	91,7
**GO:0034357**	photosynthetic membrane	cellular component	127	89,8

For each GO- ID term, the category, the number of genes identified and the percentage of down regulation revealed is reported.

**Table 3:** List of up-regulated pathogenesis-related proteins involved in tomato-*ToMV* interaction.

Protein ID	Probe ID	PRs family	Enzyme	aFold change
**Solyc01g006290.2.1**	TC197719	PR-9	Peroxidase	5.5
**Solyc01g067860.2.1**	TC195695	PR-9	Peroxidase	2.3
**Solyc01g108320.2.1**	TC209477	PR-9	Peroxidase	2.3
**Solyc02g064970.2.1**	TC201774	PR-9	Peroxidase	8.9
**Solyc02g077300.1.1**	TC205411	PR-9	Peroxidase	1.6
**Solyc02g079510.2.1**	TC196561	PR-9	Peroxidase	5.7
**Solyc02g080530.2.1**	TC209879	PR-9	Peroxidase	6.9
**Solyc02g082960.2.1**	TC200207	PR-3	Endochitinase	1.7
**Solyc02g084780.2.1**	TC201521	PR-9	Peroxidase	6.3
**Solyc02g094180.2.1**	TC211238	PR-9	Peroxidase	2.6
**Solyc03g005200.2.1**	TC203320	PR-14	Non-specific lipid-transfer protein	2.8
**Solyc03g033710.2.1**	TC203104	PR-9	Peroxidase	7.1
**Solyc03g044100.2.1**	TC199639	PR-9	Peroxidase	5.8
**Solyc04g007750.2.1**	TC199935	PR-10	Major latex-like protein	3.2
**Solyc04g071890.2.1**	TC193192	PR-9	Peroxidase	4.3
**Solyc04g072000.2.1**	TC212476	PR-3	Chitinase	1.2
**Solyc04g080760.2.1**	TC206568	PR-9	Peroxidase	2.1
**Solyc05g046010.2.1**	TC202014	PR-9	Peroxidase	2.4
**Solyc05g050130.2.1**	TC194980	PR-11	Acidic chitinase	2.8
**Solyc06g050440.2.1**	TC211424	PR-9	Peroxidase	6.9
**Solyc06g072220.1.1**	TC210670	PR-6	Kunitz trypsin inhibitor	8.5
**Solyc06g076630.2.1**	TC201658	PR-9	Peroxidase	7.9
**Solyc07g005380.2.1**	TC194633	PR-10	Norcoclaurine synthase	2.6
**Solyc07g009260.2.1**	TC210032	PR-12	Defensin-like protein	2.3
**Solyc07g017880.2.1**	TC209710	PR-9	Peroxidase	3.8
**Solyc07g052510.2.1**	TC208195	PR-9	Peroxidase	8.7
**Solyc08g067510.1.1**	TC213655	PR-14	Non-specific lipid-transfer protein	1.5
**Solyc09g007520.2.1**	TC200697	PR-9	Peroxidase	3.9
**Solyc09g065430.2.1**	TC214938	PR-14	Non-specific lipid-transfer protein	2.1
**Solyc09g082280.2.1**	TC209373	PR-14	Non-specific lipid-transfer protein	8.9
**Solyc10g076220.1.1**	TC209457	PR-9	Peroxidase	6.4
**Solyc11g072760.1.1**	TC215379	PR-11	Chitinase a	3.7
**Solyc12g017660.1.1**	TC198426	PR-9	Peroxidase	2.9

a Fold change is calculated using the signal log_2_ ratio.

For each entry it is reported: gene and probe ID, the PRs-family to which belongs and the description of its function.

**Table 4:** List of genes involved in carbon fixation differentially expressed during the tomato-*ToMV* interaction.

Gene ID	Function Description	Ezyme ID	Enzyme class	Up/Down-expressed
**Solyc08g013860.2.1**	Malic enzyme	ec:1.1.1.39	dehydrogenase	Up
**Solyc12g044600.2.1**	Malic enzyme	ec:1.1.1.40	dehydrogenase	Down
**Solyc03g071590.2.1**	Malate dehydrogenase	ec:1.1.1.82	dehydrogenase	Up
**Solyc04g009030.2.1**	Glyceraldehyde-3-phosphate dehydrogenase	ec:1.2.1.13	dehydrogenase	Down
**Solyc12g094640.1.1**	Glyceraldehyde-3-phosphate dehydrogenase	ec:1.2.1.13	dehydrogenase	Down
**Solyc10g018300.1.1**	Transketolase	ec:2.2.1.1	glycolaldehydetransferase	Down
**Solyc05g050970.2.1**	Transketolase	ec:2.2.1.1	glycolaldehydetransferase	Up
**Solyc08g079750.2.1**	1-aminocyclopropane-1-carboxylate synthase Aminotransferase	ec:2.6.1.1	transaminase	Down
**Solyc07g032740.2.1**	Aspartate aminotransferase	ec:2.6.1.1	transaminase	Up
**Solyc07g055210.2.1**	Aspartate aminotransferase	ec:2.6.1.1	transaminase	Up
**Solyc11g044840.1.1**	LL-diaminopimelate aminotransferase	ec:2.6.1.1	transaminase	Up
**Solyc05g013380.2.1**	Alanine aminotransferase	ec:2.6.1.2	transaminase	Down
**Solyc06g063090.2.1**	Alanine aminotransferase	ec:2.6.1.2	transaminase	Up
**Solyc08g076220.2.1**	Phosphoribulokinase	ec:2.7.1.19	phosphopentokinase	Down
**Solyc04g008740.2.1**	Pyruvate kinase	ec:2.7.1.40	kinase	Up
**Solyc07g066600.2.1**	Phosphoglycerate kinase	ec:2.7.2.3	kinase	Down
**Solyc07g066610.2.1**	Phosphoglycerate kinase	ec:2.7.2.3	kinase	Down
**Solyc09g011810.2.1**	Fructose-1 6-bisphosphatase	ec:3.1.3.11	hexose diphosphatase	Down
**Solyc10g086730.1.1**	Fructose-1 6-bisphosphatase	ec:3.1.3.11	hexose diphosphatase	Down
**Solyc12g014250.1.1**	Phosphoenolpyruvate carboxylase	ec:4.1.1.31	carboxylase	Down
**Solyc10g007290.2.1**	Phosphoenolpyruvate carboxylase	ec:4.1.1.31	carboxylase	Up
**Solyc01g007330.2.1**	Ribulose bisphosphate carboxylase	ec:4.1.1.39	carboxylase	Down
**Solyc03g034220.2.1**	Ribulose bisphosphate carboxylase	ec:4.1.1.39	carboxylase	Down
**Solyc06g061280.2.1**	Cinnamoyl-CoA reductase-like protein	ec:4.1.1.49	carboxykinase	Up
**Solyc01g110360.2.1**	Fructose-bisphosphate aldolase	ec:4.1.2.13	aldolase	Down
**Solyc07g065900.2.1**	Fructose-bisphosphate aldolase	ec:4.1.2.13	aldolase	Down
**Solyc09g009260.2.1**	Fructose-bisphosphate aldolase	ec:4.1.2.13	aldolase	Up
**Solyc10g083570.1.1**	Fructose-bisphosphate aldolase	ec:4.1.2.13	aldolase	Up
**Solyc03g115820.2.1**	Ribulose-5-phosphate-3-epimerase	ec:5.1.3.1	epimerase	Down
**Solyc06g005490.2.1**	Triosephosphate	ec:5.3.1.1	isomerase	Down

For each gene is reported: the functional description, the enzyme ID, the enzyme class and direction of expression regulation.

**Table 5:** List of the gibberellin modulated genes, up-regulated in tomato-*ToMV* interaction.

Gene ID	Probe ID	Putative function	GO-ID term
**Solyc02g083880.2.1**	TC203352	Gibberellin-regulated protein	GO:0005515
**Solyc06g008870.2.1**	TC202708	Gibberellin receptor	GO:0005515; GO:0008152
**Solyc06g035530.2.1**	TC202928	Gibberellin 20-oxidase-2	GO:0016491; GO:0045544; GO:0055114
**Solyc10g005360.2.1**	TC204547	Gibberellin 2-beta-dioxygenase	GO:0016491,GO:0045543
**Solyc10g050880.1.1**	TC198803	Gibberellin receptor	GO:0004091,GO:0008152
**Solyc11g072310.1.1**	TC211262	Gibberellin 20-oxidase-3	GO:0016491,GO:0045544,GO:0055114

For each gene is reported: genes and probe ID, the description of putative function and the GO-ID terms correlated.

### Relationship between Genomics and Transcriptional Changes

**Figure 4:**
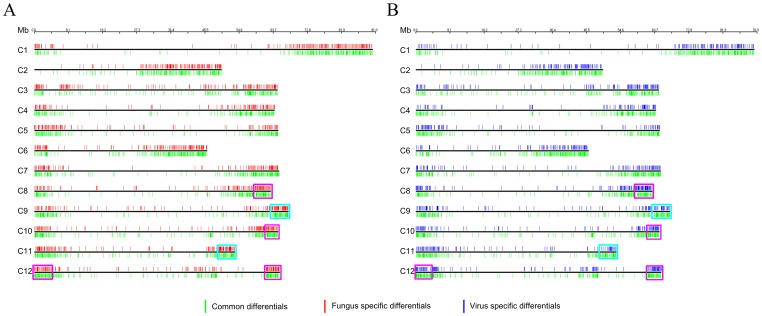
Physical genome locations of tomato (*Solanum lycopersicum*) differentially regulated genes. **A**, Physical map of the tomato-*Fol* interaction. **B**, Physical map of the tomato-*ToMV* interaction. Tomato chromosomes are represented as black horizontal bars, the approximate location of each gene is designated with vertical lines on chromosomes. The color used for each gene indicates specificity (red: differentially regulated genes in tomato-fungus interaction, blue: differentially regulated genes in tomato-virus interaction, green: overlapping differentially regulated genes. The cyan boxes on chromosomes 9 and 11 indicates the chromosomal regions in the vicinity of the *Tm2(2)* and *I2* genes. The violet boxes on chromosomes 8, 10 and 12 indicates the richest regions of genes.

In order to investigate the chromosomal arrangement of differential expressed sequences identified by tomato chip, a genomic expression map was constructed (Fig. 4). Our results indicated that differentially expressed genes (about 33% of all genes analyzed) during the two interactions were arranged along the chromosomes with different levels of expression. The fungus and virus distribution showed slight differences. Indeed, the number of expressed genes identified for single chromosomes was always greater in tomato-*ToMV* interaction than in tomato-*Fol* interaction, with the exception of chromosome 8. Chromosomal areas (on chromosomes 1, 2, 8, 11 and 12) showed a higher density of up-regulated genes in the fungus-interaction. By contrast, on chromosome 8 we found a region with a higher density of down-regulated genes in the virus-interaction. In both interactions, the genomic regions rich in differentially expressed genes were located in the vicinity of telomeres, *inter alia* the richest regions of genes were the long arm of chromosomes 10 and 12.

**Figure 5:**
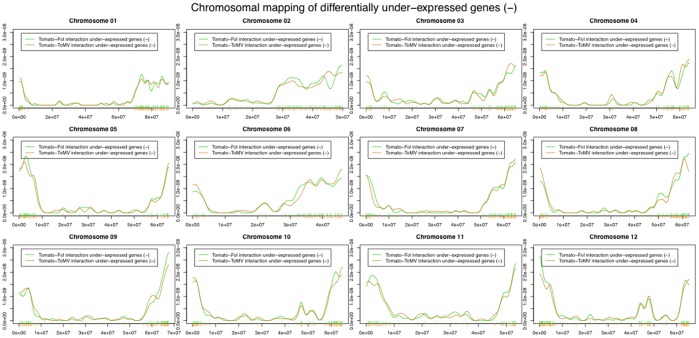
Distribution of chromosome location of under-expressed genes. The x-axis in each panel represents the range of the corresponding chromosome. The location of each differentially regulated genes in each chromosome is represented as a vertical lines at the midpoint of the location of the corresponding base pairs. The distribution of the location of all the coding genes spotted on the microarray is used as a reference and is drawn in red. The orange line represents the distribution of under-expressed genes in tomato-*ToMV* interaction. The green line represents the distribution of under-expressed genes in tomato-*Fol* interaction.

**Figure 6:**
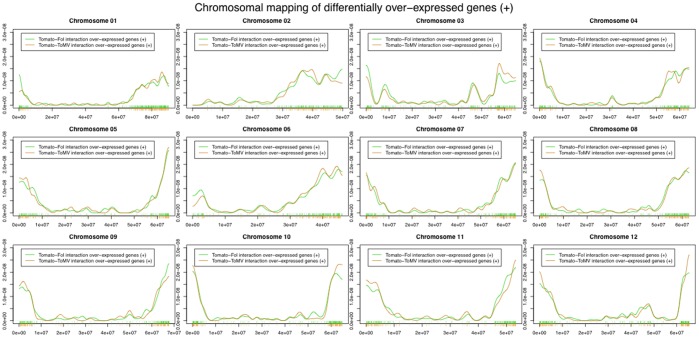
Distribution of chromosome location of over-expressed genes. The x-axis in each panel represents the range of the corresponding chromosome. The location of each differentially regulated genes in each chromosome is represented as a vertical lines at the midpoint of the location of the corresponding base pairs. The distribution of the location of all the coding genes spotted on the microarray is used as a reference and is drawn in red. The orange line represents the distribution of under-expressed genes in tomato-*ToMV* interaction. The green line represents the distribution of under-expressed genes in tomato-*Fol* interaction.

The genome-wide distribution of differentially expressed genes, based both on chromosome size and gene density was not uniformly distributed along the genome (chromosome size: fungus-interaction χ2 = 107; virus-interaction χ2 = 117. Gene density: χ2 value included between 27 and 170). Fig. 5 and 6 showed the genomic distribution of under-expressed and over-expressed genes during the two interactions. If we use the genome location of the coding genes spotted on the microarray as a reference we could highlight a significant difference in the distribution of over-expressed genes as well as under-expressed ones (Kolmogorov-Smirnov and Cramer von Mises tests in Table S3 and S4 in File S1). Indeed, differentially expressed genes (p-value <0.10) were evidenced on all chromosomes, except for chromosomes 2, 4 and 7. Stronger evidence of peculiar chromosomal displacement of over-expressed genes may be found on chromosomes 3, 5, 6 and 1 (p-value <0.05). Significantly differential displacement of under-expressed genes (p-value <0.05) was detected mainly in chromosomes 5 and 9. This suggested the presence of a dynamic genomic landscapes involved in pathogen response.

**Figure 7:**
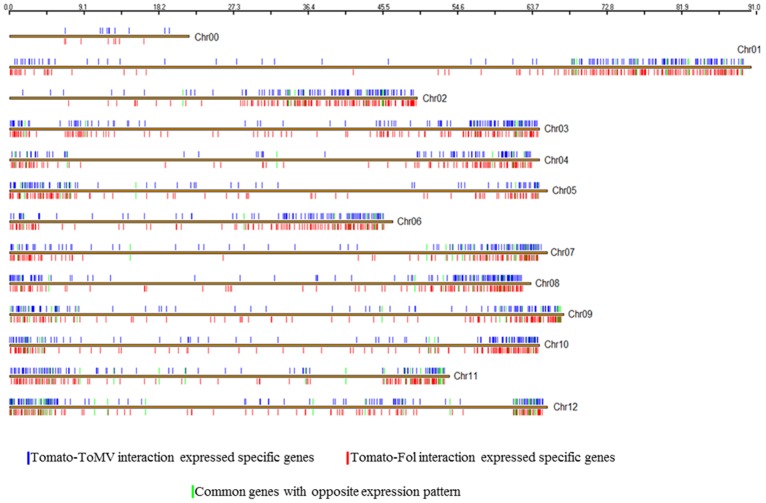
Physical map of specific differentially regulated genes in tomato-*ToMV* and tomato-*Fol* interactions. Tomato chromosomes are represented as brown horizontal bars, the approximate location of each gene is designated with vertical lines on each chromosome. The color used for each gene indicates specificity (red: differentially regulated genes in tomato-fungus interaction blue: differentially regulated genes in tomato-virus interaction, green: overlapping expressed genes in opposite direction.

**Figure 8:**
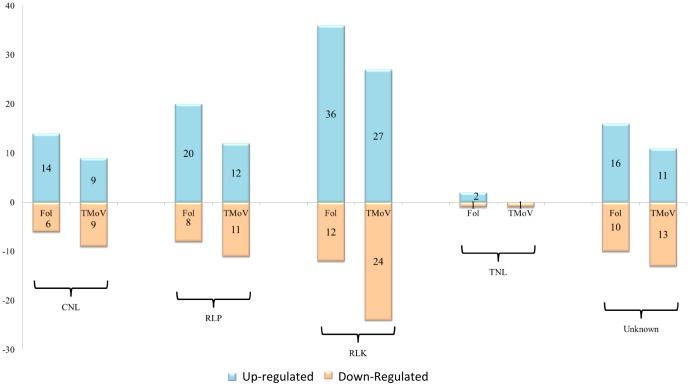
Comparison of differentially regulated tomato pathogen recognition genes during interaction with *Fusarium oxysporum (Fol)* and *Tomato Mosaic Virus (ToMV)*. Histograms display the differentially regulated pathogen recognition genes for each structural class (**NL:** NB-ARC-LRR proteins **RLP** Like Proteins; **RLK** Receptor-Like Kinases; **Unknown**: genes that encode novel domain associations or single domains) during the two incompatible interactions.

For each interaction studied, distribution of expressed specific genes is shown in Fig. 7. Most were located in the same regions, but clear differences could be highlighted. The 151 common differentially expressed genes with opposite direction were mainly arranged on chromosomes 7, 9 11 and 12. On chromosomes 9 and 11, in the vicinity of genes *Tm2(2)* conferring resistance to Tomato mosaic virus and I and I2 conferring resistance to *Fusarium oxysporum,* we highlighted differences in gene expression between the two experiments. Eleven genes were specifically expressed in *ToMV* interaction in this area of chromosome 9 (including: pentatricopeptide repeat, ATP dependent RNA helicase, cytochrome P450 and pectinesterase) and 19 of the *I2* region of chromosome 11 in *Fol* interaction (including: BEL1-like homeodomain protein 8, Calmodulin-like protein and MYB transcription factor and two proteins of unknown function).whereas around of I locus in the 1 Mb region delimitated from TG523 and CT168 [16], 8 specific genes were identified (including a Pumilio-like IPR011989 protein and Serine-threonine protein kinase). Fig. 8 shows differential expressed pathogen recognition genes (**NB-ARC-LRR**, **RLP**, **RLK**) both in tomato-*Fol* and tomato-*ToMV* interactions. Pronounced activation of all pathogen recognition gene classes assessed was evidenced in tomato-*Fol* interaction since 70% of all the differentially expressed genes were up-regulated. Instead, in the tomato-*ToMV* interaction the number of up-regulated and down-regulated pathogen recognition genes was the same: 45% of all the differentially regulated pathogen recognition genes overlapped. Of these genes, 10 had the opposite expression in the two experiments (Table S5 and S6 in File S2).

## Discussion

Plant-expressed molecules during pathogen challenging can give insights into the underlying defense mechanisms. Plants have evolved a defense system against microbial pathogens that involves the regulation of gene expression, cascade signaling activation, hormone balancing and synthesis of defensive metabolites [17]. Several microarray experiments have been successfully carried out in the sphere of molecular plant–microbe interaction, shedding light on the mechanisms controlling plant disease resistance and the crosstalk among the signaling pathways involved [4]. More than 3500 genes, out of 15,734 assessed, were activated in tomato *Fol* and *ToMV* interactions, more than 60% of which overlapped. Pathogens from different kingdoms deploy independently evolved virulence proteins that interact with a limited set of highly connected plant cellular hubs [18]. Network analysis suggests that plant response to *Fol* and *ToMV* infection involved activation or repression of genes implicated in pathways shared by most responses to environmental stimuli. However, GO enrichment analysis evidenced specific enriched categories in both interactions. Response to *ToMV* seemed more multifaceted, since more than 70 specific categories were enriched versus the 30 detected in *Fol* interaction. Biotrophic plant pathogens are generally accepted to have a more intricate biological interaction with their host plant than saprophytic plant pathogenic fungi [19]. Once an attack is perceived, plant metabolism must balance potentially competing demands for resources to support defense versus requirements for cellular maintenance, growth and reproduction [20]; [21]; [22]; [23].

In tomato-*Fol* interaction our investigation evidenced a number of overexpressed genes associated to maintenance of cellular structures and cellular homeostasis. These are very important metabolic activities required by plants to survive fungus-inflicted stresses. *Fusarium oxysporum* is a necrotrophic fungus [24] that kills host cells prior to the infection through the predicted deployment of toxins and enzymes, which induces cell death. Hence the expression of anti-apoptosis genes could confer resistance to *Fol* [25]. The NF-kB signaling enhancement found in this work can inhibit apoptosis and combat the effects of oxidative stress by switching down the anti-apoptotic signals mediated by the map-kinase cascade. These events lead us to hypothesize that the resistant plant challenged by *Fol* induces a homeostatic response to prevent the fungus from killing the cells to obtain its nourishment. Fourteen auxin-binding genes were also activated in this interaction. Auxin signaling is an important phytoregulator for resistance to saprophytic/necrotrophic fungi [26]. The degradation of AUX/IAA proteins allows activation of auxin response factors (ARFs) and the expression of auxin-responsive genes [27]. These protein families can have an enormous number of interactions, capable of fine-tuning specific responses within the auxin signaling pathway [28]. Moreover, the buildup of IAA near the sites of pathogen ingress constitutes one of the main host factors that determine plant resistance to Fusarium wilt [29]. Essential regulators of Jasmonate-ZIM domain proteins (JAZs) in tomato-*Fol* interaction were activated, suggesting attenuated JA signaling to reduce the senescence process. Indeed, also a pair of abscisic acid receptors were up-regulated in *Fol* interaction. Interactions between multiple components of ABA and the JA-ethylene signaling pathways modulate defense and stress-responsive gene expression in response to *Fol*, confirming that these hormones mediate resistance in necrothophic interaction [30]; [31]. The SA pathway proved to be switched down. Indeed, silencing a tomato gene encoding SA methyltransferase was found to enhance resistance to *F. oxysporum* f. sp. *lycopersici* biotic and abiotic stresses [32].

In tomato-*ToMV* interaction, we detected a reduced photosynthetic activity and elevated carbohydrate catabolism. Pathogen attack may have caused metabolic alterations that induced a hormone response for reprogramming the cellular metabolism. The photosynthesis and assimilatory metabolism were switched off to initiate respiration and other processes required for defense. This occurrence reflected the reallocation of plant metabolites from normal growth processes to defensive functions after the elicitation of induced plant responses by virus infection [33]. A close linkage between the plant carbohydrate-status with the resulting plant pathogen interaction was also evident. During interaction with the virus, the host produced salicylic acid and increased the production of an invertase enzyme that is able to redirect the flux of carbohydrate acquisition. The infection of *ToMV* could have led to the alteration of the concentration gradient of sucrose in phloem. The plant overexpressed β-fructofuranosidase (Solyc10g085640.1.1) to ensure the sucrose transport of source cells to the sink cells. Overexpression of invertase in tomato-virus interaction could be elicited by the expression of pathogenesis-related proteins and by salicylic acid in order to increase resistance to virus infection [34]. In order to ensure the success of defense, this mechanism appears to be crucial. The phenomenon of “high sugar resistance” was described long ago [35] and the finding that various pathogenesis-related genes were sugar-inducible [36] supports this hypothesis. We also found six gibberellin, one SAM dependent carboxyl methyltransferase (Solyc01g014320.2.1) and one jasmonate-zim domain (Solyc07g042170.2.1) genes up-regulated, suggesting that GAs activated tomato immune responses to *ToMV* by modulating the levels of salicylic acid and/or jasmonic acid [37].

Plants rely on the innate immunity and on systemic signals emanating from metabolic alterations [38]. The ETI system seems to prompt response in the right direction thanks to metabolic clues and hormone signaling. Salicylic acid (SA) primarily triggers resistance against biotrophic pathogens, whereas a combination of jasmonic acid (JA) and ethylene (ET) signaling activates resistance to necrotrophic pathogens [39]; [40]. The metabolic changes associated with defense response in two incompatible tomato-pathogen interactions suggested that the response to the specific metabolic alteration (photosynthesis/carbohydrate metabolism and homeostasis activity) in tomato was pathogen-specific and contributed substantially to monogenetic gene resistance. Our results confirm that resistance to pathogens depends on a sophisticated interplay among different biological pathways and that hormonal directionality is critical to the outcome of a response (carbohydrate biosynthesis and homeostatic activity). Several hormones involved in pathogen perception, activation of defense products restricting pathogen invasion, have been identified [2]. How they trigger ETI defense is currently unclear. In the absence of a pathogen, NB-ARC genes are in an autoinhibition state, which is relieved upon pathogen perception [41]. Although different forms of plant immunity share the same signaling mechanisms, they use the same mechanisms in very different ways [42]. The mechanisms that lead to rapid metabolism switch and connection among all the defense pathways is still not clear. Signaling able to fine-tune the defense mechanism could be activated by genes in proximity of the main pathogen receptors.

Genome organization of functional gene networks to tolerate alterations can result in plasticity. In two specific interactions the chromosome distribution of expressed genes showed wide overlapping regions, except for the region holding genes *I2* and *Tm2(2)*. Indeed, genome regions can be enriched in genes with the specific function for fine-tuning gene expression in a compensatory way. In *Fol* interaction on chromosome 11 region harboring *I2 gene* a calmodulin gene, a myb factor and a BEL-l like homeodomain protein 8 were specifically expressed. Calmodulin (CaM) plays an important role in sensing and transducing changes in cellular Ca2^+^ concentration in response to several biotic and abiotic stresses, In ToMV interaction Pentatricopeptide-repeat, ATP-dependent and RNA helicase could be involved in the process of silencing and virus replication.

A high level of NB-ARC genes activation and of other gene classes potentially involved in pathogen recognition was also found, suggesting a host-coordinated reaction of defense machinery to monitor integrity of cellular proteins. The NB-ARC system minimizes the cost of defense for the plant, as multiple NB-ARC-LRR proteins can be maintained at a low level in the absence of a pathogen and rapidly induced under pathogen attack through miRNA regulation [43]. Pathogen-encoded suppressors of RNA-silencing mechanisms might result in the induction of multiple NB-ARC-LRR defense proteins [44]. Investigation of differentially regulated pathogen recognition genes could lead to the identification of specific modulation patterns.

Albeit still fragmented, our depiction provides a global view of the *I-I2* and *Tm2(2)* mediated resistance process, in which the gene expression network may be considered the starting point to construct a genomic model for R-mediated response in the two pathosystems investigated.

## Supporting Information

File S1 Contains the following files: **Table S1.** Specific enriched GO-term categories in tomato-*Fol* interaction; **Table S2.** Specific enriched GO-term categories in tomato-ToMV interaction; **Table S3.** Results of Kolmogorov-Smirnov and Cramer von Mises analysis for testing the differences between the position distribution of **over-**expressed genes and coding genes spotted on the microarray chip; **Table S4.** Results of Kolmogorov-Smirnov and Cramer von Mises analysis for testing the differences between the position distribution of **under-**expressed genes and coding genes spotted on the microarray chip.
(DOCX)

File S2 Contains the following files: **Table S5.** Differential expressed pathogen recognition gene (NB-ARC-LRR, RLP, RLK) in tomato-Fol interaction; **Table S6.** Differential expressed pathogen recognition gene (NB-ARC-LRR, RLP, RLK) in tomato-ToMV interaction.
(XLSX)

## References

[1] Spoel SH, Dong X (2012) How do plants achieve immunity? Defense without specialized immune cells. Nature Reviews Immunology. 12: 89–100. 10.1038/nri3141 22273771

[2] López MA, Bannenberg G, Castresana C (2008) Controlling hormone signaling is a plant and pathogen challenge for growth and survival. Current Opinion in Plant Biology 11 (4): 420–427. 10.1016/j.pbi.2008.05.002 18585953

[3] Ercolano MR, Sanseverino W, Carli P, Ferriello F, Frusciante L (2012) Genetic and genomic approaches for R-gene mediated disease resistance in tomato: retrospects and prospects. Plant Cell Reports 31 (6): 973–85. 10.1007/s00299-012-1234-z PMC3351601 22350316

[4] Lodha TD, Basak J (2012) Plant-pathogen interactions: what microarray tells about it?. Molecular Biotechnology 50 (1) 87–97: 2. 10.1007/s12033-011-9418-2 21618071

[5] Inami K, Yoshioka-Akiyama C, Morita Y, Yamasaki M, Teraoka1 T, et al (2012) Emergence of Races in *Fusarium oxysporum* f. sp. *lycopersici*: Inactivation of Avirulence Gene *AVR1* by Transposon Insertion PLoS ONE 7: e44101 doi:10.1371/journal.pone.0044101 22952887 10.1371/journal.pone.0044101 PMC3428301

[6] Ori N, Eshed Y, Paran I, Presting G, Aviv D, et al (1997) The I2C family from the wilt disease resistance locus I2 belongs to the nucleotide binding, leucine-rich repeat superfamily of plant resistance genes. Plant Cell. 9 (4): 521–32. 10.1105/tpc.9.4.521 PMC156936 9144960

[7] Simons G, Groenendijk J, Wijbrandi J, Reijans M, Groenen J, et al (2012) A microRNA superfamily regulates nucleotide binding site-leucine-rich repeats and other mRNAs. Plant Cell 24(3): 859–874. 22408077 10.1105/tpc.111.095380 PMC3336131

[8] Beckman CH (2000) Phenolic-storing cells: keys to programmed cell death and periderm formation in wilt disease resistance and in general defense responses in plants? Physiological and Molecular Plant Pathology 57: 101–110.

[9] Takken F, Rep M (2010) The arms race between tomato and Fusarium oxysporum. Molecular Plant Pathology 11 (2): 309–14. 10.1111/j.1364-3703.2009.00605.x PMC6640361 20447279

[10] He M, He CQ, Ding NZ (2012) Natural recombination between tobacco and tomato mosaic viruses. Virus Research 163 (1): 374–379. 10.1016/j.virusres.2011.09.006 21925550

[11] Kobayashi M, Yamamoto-Katou A, Katou S, Hirai K, Meshi T, et al (2011) Identification of an amino acid residue required for differential recognition of a viral movement protein by the Tomato mosaic virus resistance gene Tm-2(2). Journal of Plant Physiology 168 (10): 1142–5. 10.1016/j.jplph.2011.01.002 21310506

[12] CPVO-TP/44/3 technical protocol. Community plant variety office. Protocol for distinctness, uniformity and stability tests. Lycopersicon esculentum L. Karsten ex. Farw. TOMATO. website www.cpvo.europa.eu. Accessed 2007 March 21.

[13] R Core Team (2013) R: A language and environment for statistical computing. R Foundation for Statistical Computing, Vienna, Austria. URL http://www.R-project.org/.

[14] Smyth GK (2005) Limma: linear models for microarray data. In: ‘Bioinformatics and Computational Biology Solutions using R and Bioconductor’. R. Gentleman, V. Carey, S. Dudoit, R. Irizarry, W. Huber (eds.), Springer, New York, 397–420.

[15] Smyth GK (2004) Linear models and empirical Bayes methods for assessing differential expression in microarray experiments. Stat Appl Genet Mol Biol.3. 10.2202/1544-6115.1027 16646809

[16] Sela-Buurlage MB, Budai-Hadrian O, Pan Q, Carmel- Goren L, Vunsch R, et al (2001) Mol Genet Genomics. 265: 1104–1111. 10.1007/s004380100509 11523783

[17] Mithöfer A, Boland W (2012) Plant defense against herbivores: chemical aspects. Annual Review of Plant Biology 63: 431–50. 10.1146/annurev-arplant-042110-103854 22404468

[18] Mukhtar MS, Carvunis AR, Dreze M, Epple P, Steinbrenner J, et al (2011) Independently evolved virulence effectors converge onto hubs in a plant immune system network. Science. 33 (6042): 596–601. 10.1126/science.1203659 PMC3170753 21798943

[19] Hammond-Kosack KE, Rudd JJ (2008) Plant resistance signalling hijacked by a necrotrophic fungal pathogen. Plant Signaling & Behavior 3 (11): 993–995. 10.4161/psb.6292 PMC2633754 19704431

[20] Herms DA, Mattson WJ (1992) The dilemma of plants: to grow or defend. Quarterly Review of Biology. 67: 283–335.

[21] Zangerl AR, Berenbaum MR (1997) Cost of chemically defending seeds: furanocoumarins and Pastinaca sativa. The American Naturalist 150 (4): 491–504. 10.1086/286077 18811288

[22] Zangerl AR, Berenbaum MR (2003) Phenotype matching in wild parsnip and parsnip webworms: causes and consequences. Evolution. 57(4): 806–15. 10.1111/j.0014-3820.2003.tb00292.x 12778550

[23] Berger S, Sinha AK, Roitsch T (2007) Plant physiology meets phytopathology: plant primary metabolism and plant-pathogen interactions. Journal of Experimental Botany 58 (15–16): 4019–26. 10.1093/jxb/erm298 18182420

[24] Trusov Y, Rookes JE, Chakravorty D, Armour D, Schenk PM, et al (2006) Heterotrimeric G proteins facilitate Arabidopsis resistance to necrotrophic pathogens and are involved in jasmonate signaling. Plant Physiology 140 (1): 210–220. 10.1104/pp.105.069625 PMC1326045 16339801

[25] Paul JY, Becker DK, Dickman MB, Harding RM, Khanna HK, et al (2011) Apoptosis-related genes confer resistance to Fusarium wilt in transgenic ‘Lady Finger’ bananas. Journal of Plant Biotechnology 9 (9): 1141–1148. 10.1111/j.1467-7652.2011.00639.x 21819535

[26] Llorente F, Muskett P, Sánchez-Vallet A, López G, Ramos B, et al (2008) Repression of the auxin response pathway increases Arabidopsis susceptibility to necrotrophic fungi. Molecular Plant 1 (3): 496–509. 10.1093/mp/ssn025 19825556

[27] Hagen G, Guilfoyle T (2002) Auxin-responsive gene expression: genes, promoters and regulatory factors. Plant Molecular Biology 49 (3–4): 373–85. 12036261

[28] Guilfoyle TJ, Hagen G (2007) Auxin response factors Current Opinion in Plant Biolology 10. (5): 453–60. 10.1016/j.pbi.2007.08.014 17900969

[29] Dalila Paz-Lago A, Borges Jr A, Gutiérrez A, Borges G, Cabrera MA, et al (2000) Tomato Fusarium oxysporum interactions:ii-chitosan and msb induced resistance against fol in young tomato plants. Cultivos Tropicales 21 (4): 17–20.

[30] Badruzsaufari E, Schenk PM, Manners JM, Desmond OJ, Ehlert C, et al (2004) Antagonistic interaction between abscisic acid and jasmonate-ethylene signaling pathways modulates defense gene expression and disease resistance in Arabidopsis. Plant Cell. 16 (12): 3460–3479. 10.1105/tpc.104.025833 PMC535886 15548743

[31] Koornneef A, Verhage A, Leon-Reyes A, Snetselaar R, Van Loon L, et al (2008) Towards a reporter system to identify regulators of cross-talk between salicylate and jasmonate signaling pathways in Arabidopsis. Plant Signaling & Behavior 3 (8): 543–546. 10.4161/psb.3.8.6151 PMC2634489 19513248

[32] Ament K, Krasikov V, Allmann S, Rep M, Takken FL, et al (2010) Methylsalicylateproduction in tomato affects biotic interactions. The Plant Journal. 62: 124–34. 10.1111/j.1365-313X.2010.04132.x 20059742

[33] Handford MG, Carr JP (2007) A defect in carbohydrate metabolism ameliorates symptom severity in virus-infected Arabidopsis thaliana. Journal of General Virology 88 (1): 337–41. 10.1099/vir.0.82376-0 17170466

[34] Herbers K, Takahata Y, Melzer M, Mock HP, Hajirezaei M, et al (2000) Regulation of carbohydrate partitioning during the interaction of potato virus Y with tobacco. Molecular Plant Pathology 1 (1): 51–59. 10.1046/j.1364-3703.2000.00007.x 20572950

[35] Horsfall JG, Diamond AE (1957) The diseased plant in plant pathology - An advanced treatise. Academic Press New York. 5: 1–17.

[36] Herbers K, Meuwly P, Métraux JP, Sonnewald U (1996) Salicylic acid-independent induction of pathogenesis-related protein transcripts by sugars is dependent on leaf developmental stage. FEBS Letters 397 (2–3): 239–44. 10.1016/s0014-5793(96)01183-0 8955355

[37] de Torres Zabala M, Bennett MH, Truman WH, Grant MR (2009) Antagonism between salicylic and abscisic acid reflects early host-pathogen conflict and moulds plant defence responses. The Plant Journal 59 (3): 375–386. 10.1111/j.1365-313X.2009.03875.x 19392690

[38] Jones JD, Dangl JL (2006) The plant immune system. Nature. 444: 323–329. 10.1038/nature05286 17108957

[39] Glazebrook J (2005) Contrasting mechanisms of defense against biotrophic and necrotrophic pathogens. Annual Review of Phytopathology 43: 205–227. 10.1146/annurev.phyto.43.040204.135923 16078883

[40] Robert-Seilaniantz A, MacLean D, Jikumaru Y, Hill L, Yamaguchi S, et al (2011) The microRNA miR393 re-directs secondary metabolite biosynthesis away from camalexin and towards glucosinolates. The Plant Journal. 67: 218–231. 10.1111/j.1365-313X.2011.04591.x 21457368

[41] Lukasik E, Takken FL (2009) STANDing strong, resistance proteins instigators of plant defense. Current Opinion in Plant Biology 12 (4): 427–436. 10.1016/j.pbi.2009.03.001 19394891

[42] Tsuda K, Sato M, Stoddard T, Glazebrook J, Katagiri F (2009) Network Properties of Robust Immunity in Plants. PLoS Genetics 5 (12): e1000772. 10.1371/journal.pgen.1000772 PMC2782137 20011122

[43] Li J, Zhang W, Wu H, Guo T, Liu X, et al (2011) Fine mapping of stable QTLs related to eating quality in rice (*Oryza sativa* L.) by CSSLs harboring small target chromosomal segments. Breeding Science 61 (4): 338–46. 10.1270/jsbbs.61.338 PMC3406775 23136470

[44] Shivaprasad P, Chen HM, Patel K, Bond DM, Santos BA, et al (2012) A microRNA superfamily regulates nucleotide binding site-leucine-rich repeats and other mRNAs. Plant Cell 24(3): 859–874. 22408077 10.1105/tpc.111.095380 PMC3336131

