# Supplementary material for: Tomato Genome-Wide Transcriptional Responses to Fusarium Wilt and Tomato Mosaic Virus
Source: PLoS One. 2014 May 7;9(5):e94963. doi: 10.1371/journal.pone.0094963 (PMC4012952; doi:10.1371/journal.pone.0094963)
Supplement: File S1 — Contains the following files: Table S1. Specific enriched GO-term categories in tomato-Fol interaction; Table S2. Specific enriched GO-term categories in tomato-ToMV interaction; Table S3. Results of Kolmogorov-Smirnov and Cramer von Mises analysis for testing the differences between the position distribution of over-expressed genes and coding genes spotted on the microarray chip; Table S4. Results of Kolmogorov-Smirnov and Cramer von Mises analysis for testing the differences between the position distribution of under-expressed genes and coding genes spotted on the microarray chip. (DOCX) [file pone.0094963.s001.docx]

**Table S1.** Specific enriched GO-term categories in tomato-*Fol* interaction.

| GO ID | Term description |
| --- | --- |
| GO:0010304 | PSII associated light-harvesting complex II catabolic process |
| GO:0007276 | gamete generation |
| GO:0010155 | regulation of proton transport |
| GO:0009543 | chloroplast thylakoid lumen |
| GO:0031978 | plastid thylakoid lumen |
| GO:0010114 | response to red light |
| GO:0016226 | iron-sulfur cluster assembly |
| GO:0031163 | metallo-sulfur cluster assembly |
| GO:0009073 | aromatic amino acid family biosynthetic process |
| GO:0010218 | response to far red light |
| GO:0046417 | chorismate metabolic process |
| GO:0043648 | dicarboxylic acid metabolic process |
| GO:0009746 | response to hexose stimulus |
| GO:0010103 | stomatal complex morphogenesis |
| GO:0034284 | response to monosaccharide stimulus |
| GO:0006873 | cellular ion homeostasis |
| GO:0010374 | stomatal complex development |
| GO:0005783 | endoplasmic reticulum |
| GO:0055080 | cation homeostasis |
| GO:0009072 | aromatic amino acid family metabolic process |
| GO:0050801 | ion homeostasis |
| GO:0019725 | cellular homeostasis |
| GO:0042743 | hydrogen peroxide metabolic process |
| GO:0048878 | chemical homeostasis |
| GO:0072593 | reactive oxygen species metabolic process |
| GO:0010016 | shoot morphogenesis |
| GO:0042592 | homeostatic process |
| GO:0016053 | organic acid biosynthetic process |
| GO:0046394 | carboxylic acid biosynthetic process |
| GO:0065008 | regulation of biological quality |

**Table S2.** Specific enriched GO-term categories in tomato-ToMV interaction.

| GO ID | Term description |
| --- | --- |
| GO:0016020 | membrane |
| GO:1901135 | carbohydrate derivative metabolic process |
| GO:0044271 | cellular nitrogen compound biosynthetic process |
| GO:0016051 | carbohydrate biosynthetic process |
| GO:0032991 | macromolecular complex |
| GO:0030529 | ribonucleoprotein complex |
| GO:0051188 | cofactor biosynthetic process |
| GO:0044249 | cellular biosynthetic process |
| GO:0051179 | localization |
| GO:0043228 | non-membrane-bounded organelle |
| GO:0051234 | establishment of localization |
| GO:0010038 | response to metal ion |
| GO:0015934 | large ribosomal subunit |
| GO:0006950 | response to stress |
| GO:0006094 | gluconeogenesis |
| GO:0009853 | photorespiration |
| GO:0019748 | secondary metabolic process |
| GO:0009117 | nucleotide metabolic process |
| GO:0006952 | defense response |
| GO:0006412 | translation |
| GO:0022625 | cytosolic large ribosomal subunit |
| GO:0046686 | response to cadmium ion |
| GO:0018298 | protein-chromophore linkage |
| GO:0015977 | carbon fixation |
| GO:0009058 | biosynthetic process |
| GO:0051704 | multi-organism process |
| GO:0015935 | small ribosomal subunit |
| GO:0019843 | rRNA binding |
| GO:0030001 | metal ion transport |
| GO:0042170 | plastid membrane |
| GO:0006820 | anion transport |
| GO:0005840 | ribosome |
| GO:0022626 | cytosolic ribosome |
| GO:0071555 | cell wall organization |
| GO:0005730 | nucleolus |
| GO:0006790 | sulfur compound metabolic process |
| GO:0044445 | cytosolic part |
| GO:0005829 | cytosol |
| GO:0006753 | nucleoside phosphate metabolic process |
| GO:0009056 | catabolic process |
| GO:0048046 | apoplast |
| GO:0005576 | extracellular region |
| GO:0005618 | cell wall |
| GO:0043094 | cellular metabolic compound salvage |
| GO:0006096 | glycolysis |
| GO:0019685 | photosynthesis, dark reaction |
| GO:0005507 | copper ion binding |
| GO:0042214 | terpene metabolic process |
| GO:0010035 | response to inorganic substance |
| GO:0032787 | monocarboxylic acid metabolic process |
| GO:0016491 | oxidoreductase activity |
| GO:0016556 | mRNA modification |
| GO:0031969 | chloroplast membrane |
| GO:0018130 | heterocycle biosynthetic process |
| GO:0044391 | ribosomal subunit |
| GO:0046688 | response to copper ion |
| GO:0046364 | monosaccharide biosynthetic process |
| GO:0019253 | reductive pentose-phosphate cycle |
| GO:0019319 | hexose biosynthetic process |
| GO:0016119 | carotene metabolic process |
| GO:0003735 | structural constituent of ribosome |
| GO:0016829 | lyase activity |
| GO:0044272 | sulfur compound biosynthetic process |
| GO:0000271 | polysaccharide biosynthetic process |
| GO:0005975 | carbohydrate metabolic process |
| GO:0034637 | cellular carbohydrate biosynthetic process |
| GO:0005198 | structural molecule activity |
| GO:0009773 | photosynthetic electron transport in photosystem I |
| GO:0016168 | chlorophyll binding |
| GO:0030312 | external encapsulating structure |
| GO:0043232 | intracellular non-membrane-bounded organelle |

**Table S3.** Results of Kolmogorov-Smirnov and Cramer von Mises analysis for testing the differences between the position distribution of **over-**expressed genes and coding genes spotted on the microarray chip (Choulakian 1994; Conover 1972)

| **chrom** | **Fol.ks** | **Fol.cvm** | **ToMV.ks** | **ToMV.cvm** |
| --- | --- | --- | --- | --- |
| 01 | 0.753 | 0.617 | 0.290 | *0.083* |
| 02 | 0.597 | 0.660 | 0.711 | 0.509 |
| 03 | **0.024** | *0.069* | 0.163 | 0.275 |
| 04 | 0.885 | 0.762 | 0.634 | 0.678 |
| 05 | *0.078* | **0.027** | 0.202 | *0.075* |
| 06 | 0.322 | 0.260 | **0.048** | **0.022** |
| 07 | 0.812 | 0.874 | 0.940 | 0.894 |
| 08 | 0.715 | 0.925 | 0.764 | 0.816 |
| 09 | 0.293 | 0.356 | 0.632 | 0.561 |
| 10 | 0.851 | 0.592 | 0.123 | *0.099* |
| 11 | **0.011** | **0.031** | 0.816 | 0.610 |
| 12 | 0.460 | 0.315 | 0.510 | 0.376 |

**Table S4.** Results of Kolmogorov-Smirnov and Cramer von Mises analysis for testing the differences between the position distribution of **under-**expressed genes and coding genes spotted on the microarray chip (Choulakian 1994; Conover,1972)*.

| **chrom** | **Fol.ks** | **Fol.cvm** | **ToMV.ks** | **ToMV.cvm** |
| --- | --- | --- | --- | --- |
| 01 | 0.650 | 0.578 | 0.828 | 0.869 |
| 02 | 0.531 | 0.403 | 0.341 | 0.281 |
| 03 | 0.501 | 0.655 | *0.060* | 0.130 |
| 04 | 0.731 | 0.881 | 0.978 | 0.934 |
| 05 | **0.048** | *0.052* | 0.128 | 0.122 |
| 06 | 0.799 | 0.724 | 0.982 | 0.943 |
| 07 | 0.806 | 0.733 | 0.288 | 0.144 |
| 08 | 0.135 | *0.050* | *0.086* | 0.105 |
| 09 | *0.070* | **0.045** | 0.534 | 0.554 |
| 10 | 0.782 | 0.898 | 0.993 | 0.955 |
| 11 | 0.363 | 0.510 | 0.216 | 0.367 |
| 12 | 0.161 | *0.097* | 0.752 | 0.648 |

* Choulakian, V. Lockhart, R. A. and Stephens, M. A. 1994. Cramer-von Mises statistics for discrete distributions. Can J Stat. 22(1): 125-137. Conover, W. J. 1972. A Kolmogorov Goodness-of-Fit Test for Discontinuous Distributions. J Am Statist. Ass. 67 (339) 591–596.
